# GART Functions as a Novel Methyltransferase in the RUVBL1/β‐Catenin Signaling Pathway to Promote Tumor Stemness in Colorectal Cancer

**DOI:** 10.1002/advs.202301264

**Published:** 2023-07-13

**Authors:** Chao Tang, Mengying Ke, Xichao Yu, Shanliang Sun, Xian Luo, Xin Liu, Yanyan Zhou, Ze Wang, Xing Cui, Chunyan Gu, Ye Yang

**Affiliations:** ^1^ Nanjing Drum Tower Hospital Clinical College of Traditional Chinese and Western Medicine Nanjing University of Chinese Medicine Nanjing 210008 China; ^2^ School of Medicine & Holistic Integrative Medicine Nanjing University of Chinese Medicine Nanjing 210046 China; ^3^ School of Pharmacy Nanjing University of Chinese Medicine Nanjing 210046 China; ^4^ Department of Hematology and Oncology The Second Affiliated Hospital of Shandong University of Traditional Chinese Medicine Jinan 250001 China

**Keywords:** cancer stem cells, colorectal cancer, glycinamide ribonucleotide transformylase, pemetrexed, RUVBL1/β‐catenin axis

## Abstract

Tumor stemness is associated with the recurrence and incurability of colorectal cancer (CRC), which lacks effective therapeutic targets and drugs. Glycinamide ribonucleotide transformylase (GART) fulfills an important role in numerous types of malignancies. The present study aims to identify the underlying mechanism through which GART may promote CRC stemness, as to developing novel therapeutic methods. An elevated level of *GART* is associated with poor outcomes in CRC patients and promotes the proliferation and migration of CRC cells. CD133^+^ cells with increased GART expression possess higher tumorigenic and proliferative capabilities both in vitro and in vivo. GART is identified to have a novel methyltransferase function, whose enzymatic activity center is located at the E948 site. GART also enhances the stability of RuvB‐like AAA ATPase 1 (RUVBL1) through methylating its K7 site, which consequently aberrantly activates the Wnt/β‐catenin signaling pathway to induce tumor stemness. Pemetrexed (PEM), a compound targeting GART, combined with other chemotherapy drugs greatly suppresses tumor growth both in a PDX model and in CRC patients. The present study demonstrates a novel methyltransferase function of GART and the role of the GART/RUVBL1/β‐catenin signaling axis in promoting CRC stemness. PEM may be a promising therapeutic agent for the treatment of CRC.

## Graphical abstract

Glycinamide ribonucleotide transformylase (GART) is identified to have a novel methyltransferase activity with an enzyme activity center at the E948 site, which prompts RUVBL1 K7 site methylation and inhibits ubiquitination degradation of RUVBL1 to promote colorectal cancer (CRC) stemness via activating the RUVBL1/β‐catenin axis. Translationally, pemetrexed, a selective GART inhibitor, combined with CRC chemotherapy drugs significantly suppresses tumor growth and prolongs patient survival.

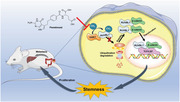

## 1 Introduction

Colorectal cancer (CRC) is the third most common malignant tumor worldwide, accounting for approx. 10% of all cases of diagnosed cancer and cancer‐associated deaths per year.^[^ ^1^, ^2^ ^]^ Advanced treatments with chemotherapeutic drugs can prolong patient survival, including the administration of oxaliplatin, fluorouracil and capecitabine;^[^ ^3^, ^4^, ^5^ ^]^ however, certain patients with CRC will ultimately develop relapses. Notably, accumulating evidence has revealed that cancer stem cells of CRC (CCSCs) are responsible for the development, recurrence and chemotherapeutic resistance of CRC.^[^ ^6^, ^7^ ^]^

CCSCs, with the capabilities of self‐renewal, high heterogeneity and metastatic potential, are able to promote CRC progression.^[^ ^8^, ^9^, ^10^, ^11^ ^]^ CD133 is a biomarker of CCSCs, and it has been reported that CD133^+^ CRC cells are more prone to metastasis compared with CD133^‐^ CRC cells.^[^ ^12^ ^]^ Several studies have suggested that using CD133 as a target for antibody neutralization therapy, or in combination with other chemotherapeutic drugs, confers satisfying therapeutic effects on CRC, at least to a certain extent.^[^ ^13^, ^14^, ^15^ ^]^ Unfortunately, the development of CD133 antibodies to treat CRC remains problematic, and at the present time has not reached a suitable stage for the clinic.^[^ ^16^, ^17^, ^18^, ^19^ ^]^ Therefore, there is an urgency to further develop therapeutic methods utilizing CCSCs. CCSCs are regulated via aberrant activation of the Wnt/β‐catenin, Notch and TGF‐β signaling pathways.^[^ ^20^, ^21^ ^]^ The Wnt/β‐catenin signaling pathway exerts a key role in regulating the physiological functions and homeostasis of normal intestinal stem cells^[^ ^22^, ^23^, ^24^ ^]^; however, aberrant activation of the Wnt/β‐catenin signaling pathway drives the initiation of CRC, and promotes the maintenance and development of CRC via CCSCs,^[^ ^25^, ^26^ ^]^ indicating that targeting the Wnt/β‐catenin axis may be an alternative method for treating CCSCs.

It has been reported that arsenic trioxide (ATO) is a small‐molecule drug whose application in the treatment of acute promyelocytic leukemia (APL) not only promotes tumor cell differentiation and apoptosis, but also effectively eliminates APL stem cells.^[^ ^27^, ^28^, ^29^, ^30^, ^31^ ^]^ ATO has also been utilized to treat certain types of solid tumors, including colorectal cancer, hepatocellular cancer, lung cancer, glioblastoma, and so on, through targeting CSCs.^[^ ^32^, ^33^, ^34^, ^35^, ^36^, ^37^ ^]^ Our research group previously analyzed the proteome microarray experiments performed by Zhang et al. and demonstrated that glycinamide ribonucleotide transformylase (GART) binds tightly to ATO.^[^ ^38^ ^]^ GART is a key folate coenzyme in the de novo synthesis pathway that catalyzes the transformation of its substrate glycinamide nucleotides into formylglycinamide nucleotides, so as to influence the formation of nucleotides for DNA replication.^[^ ^39^, ^40^ ^]^ GART is highly expressed in certain types of solid tumor, such as liver cancer and glioma, including CRC,^[^ ^41^, ^42^ ^]^ which suggests that GART may be closely associated with the development of various malignancies.

The aim of the present study was to explore the novel methyltransferase function of GART and mechanisms in regulating CRC stemness. In addition, as a compound targeting the methyltransferase center of GART, pemetrexed (PEM) in combination with chemotherapy drugs were investigated for the therapeutic effects both on CRC patient‐derived xenograft (PDX)‐modeled mice and patients in a clinical setting.

## 2 Results

### 2.1 Elevated Levels of *GART* are Associated with Poor Survival in Patients with CRC, and Promote CRC Cell Proliferation in vitro and in vivo

**Figure 1:**
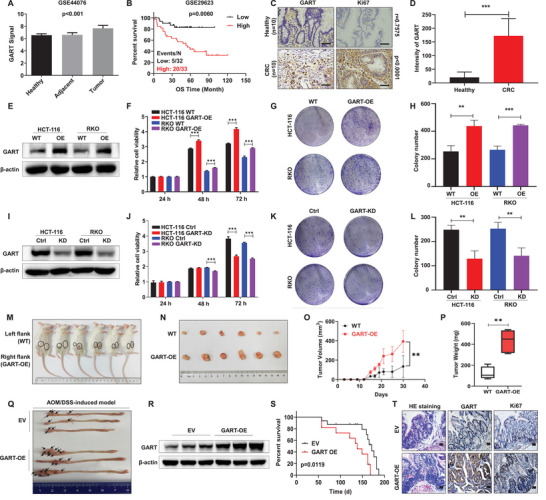
Elevated levels of GART are associated with poor survival in patients with CRC, and promote CRC cell proliferation and tumorigenicity both in vitro and in vivo. A) The mRNA expression level of GART in patients with colorectal cancer (CRC) (n = 98) from the GSE44076 database was higher compared with that in healthy (n = 50) or adjacent tissues (n = 98). B) An increased level of GART was significantly correlated with poor overall survival (OS) in the GSE29623 cohort. C) Representative IHC staining of healthy (n = 10) and CRC (n = 10) samples. Scale bar = 50 µm. D) IHC staining intensity analyses of GART in healthy and CRC groups. E) WB was performed to detect GART expression in WT and GART‐OE CRC cells. F) CCK8 assay tested the proliferation capacity in WT and GART‐OE CRC cells. G) Colony formation assay examined the long‐term proliferation of WT and GART‐OE CRC cells. H) A histogram showing the statistical results of colony formation assay in WT and GART‐OE CRC cells. I) WB was used to determine the expression of GART in GART‐KD CRC cells upon induction with tetracycline. J) CCK8 assay showing the proliferative ability of control (Ctrl) and GART‐KD CRC cells. K) Colony formation assay examining the long‐term proliferation of Ctrl and GART‐KD CRC cells. L) The histogram showed the statistical results of colony formation assay in Ctrl and GART‐KD CRC cells. M) Photographic images of xenograft mice at Day 30. WT and GART‐OE HCT‐116 cells were injected subcutaneously into NOD/SCID mice. N) Schematic images of xenograft tumors from 6 NOD/SCID mice. O) Time course of tumor growth in 6 NOD/SCID mice. P) Mean tumor weight in 6 NOD/SCID mice. Q) Macroscopic appearance of the tumors in the large intestines from azoxymethane (AOM)/dextran sulfate sodium (DSS)‐induced BALB/c mice. R) WB analysis showing the expression of GART in AOM/DSS‐induced tumor tissues. S) Kaplan−Meier survival curve analysis of the BALB/c mice treated with AOM/DSS. T) Representative images of H&E and immunohistochemistry (IHC) staining of indicated targets on tumor sections. Scale bar = 50 µm. The data are expressed as the mean ± SD. **p<0.01, ***p<0.001.

First, we examined the expression of *GART* in the Gene Expression Omnibus (GEO) databases, and found that the level of *GART* was significantly increased in CRC samples compared with the adjacent healthy tissues (**Figure** **1**A). Kaplan‐Meier analysis indicated that CRC patients with an elevated level of *GART* suffered from poor survival (Figure 1B). In agreement with the above results, immunohistochemistry (IHC) assay demonstrated that GART was strongly expressed in CRC samples compared with the healthy tissues. As a vital marker of cell proliferation, Ki67 was found to be positively correlated with GART expression (*p*<0.0001; *r* = 0.7575) (Figure 1C,D). In addition, GART expression was significantly correlated with pathological grade, lymph node metastasis, distant metastasis and survival status, but not with age, gender and tumor site (**Table** **1**).

**Table 1:** Correlation of GART expression and clinicopathologic characteristics of CRC patients

		GART expression [%]			
Characteristic	n	High	Low or no	Pearson χ^2^	*P*
Total	119	58 [48.74]	61 [51.26]		
Age				0.943	NS
≥ 60	74	33 [44.59]	41 [55.41]		
< 60	45	25 [55.56]	20 [44.44]		
Gender				0.112	NS
Female	48	22 [45.83]	26 [54.17]		
Male	71	36 [50.70]	35 [49.30]		
Tumor site				0.343	NS
Colon	41	22 [53.66]	19 [46.34]		
Rectum	78	36 [46.15]	42 [53.85]		
Pathological grade				11.833	<0.01
I‐II	75	27 [36.00]	48 [64.00]		
III‐IV	44	31 [70.45]	13 [29.55]		
Lymph node metastasis				8.415	<0.01
No	78	30 [38.46]	48 [61.54]		
Yes	41	28 [68.29]	13 [31.71]		
Distant metastasis				4.945	<0.05
No	107	48 [44.86]	59 [55.14]		
Yes	12	10 [83.33]	2 [16.67]		
Survival status				3.960	<0.05
Death	16	12 [75.00]	4 [25.00]		
Survival	103	46 [44.66]	57 [55.34]		

Subsequently, the functional role of GART in CRC cell lines was further investigated. The stable overexpression of GART (GART‐OE) in HCT‐116 and RKO CRC cell lines was confirmed by western blot (WB) analysis (Figure 1E). CCK8 assays showed that an elevated level of GART significantly promoted the proliferation of both HCT‐116 and RKO cells (Figure 1F). Furthermore, colony formation assays also indicated that a high expression level of GART could increase the long‐term proliferative capabilities of HCT‐116 and RKO cells (Figure 1G,H). Conversely, HCT‐116 and RKO cells were transfected with GART lentiviral shRNA particles, after which the knockdown efficiency was validated by WB analysis (Figure 1I). The cell proliferation rate was found to be significantly decreased in GART knockdown (GART‐KD) cells compared with control (Ctrl) cells (Figure 1J–L).

Next, two xenograft tumor models and an azoxymethane (AOM)/dextran sulfate sodium (DSS)‐induced CRC model were used, respectively, to further explore whether GART could promote CRC tumor growth in vivo. WT and GART‐OE HCT‐116 cells were injected subcutaneously into 6 NOD/SCID mice and 6 nude mice, respectively. As shown in Figure 1M,N, the tumors formed by the GART‐OE cells were visibly larger compared with their WT counterparts in NOD/SCID mice. Moreover, an elevated level of GART led to a marked acceleration in the growth rate of xenograft tumors in NOD/SCID mice (Figure 1O,P). Interestingly, the injection of WT HCT‐116 cells led to tumor formation in only one nude mouse, which may have been attributable to the presence of retained immunity in the nude mice (Figure S1, Supporting Information). By contrast, GART‐OE HCT‐116 cells caused tumor formation in all 6 nude mice, indicating that GART strengthened the tumor formation capacity in vivo. Since adeno‐associated virus 9 (AAV9) could effectively and stably achieve gene transduction in intestinal epithelial cells,^[^ ^43^, ^44^ ^]^ 4‐week‐old male BALB/c mice were transduced with AAV9‐GART overexpression (AAV9‐GART‐OE) or control (AAV9‐EV) vectors via tail vein injection. After 2 weeks, AOM/DSS administration was performed (Figure S2, Supporting Information). These experiments revealed that the tumor growth in the colorectal intestine of AAV9‐GART‐OE mice was faster compared with that in the AAV9‐EV mice (Figure 1Q). WB analysis confirmed that GART expression was increased in the colorectal tissue samples of AAV9‐GART‐OE mice compared with AAV9‐EV mice (Figure 1R). Kaplan‐Meier survival curve analysis subsequently showed that the survival times of the AAV9‐GART‐OE mice were significantly prolonged compared with those of the AAV9‐EV mice (Figure 1S). Moreover, the analyses of IHC and H&E staining revealed that the expression levels of GART were higher at the colorectal site and in the tumor tissues in AAV9‐GART‐OE mice compared with the AAV9‐EV mice (Figure 1T). Collectively, these findings suggest that a high expression level of GART is associated with poor prognosis in patients with CRC and promotes CRC cell proliferation both in vivo and in vitro.

### 2.2 GART Contributes to CRC Cell Migration in vitro and in vivo

**Figure 2:**
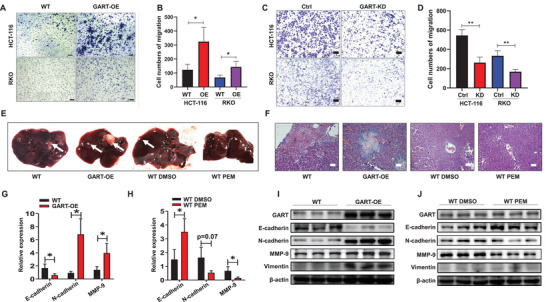
Increased levels of GART promotes CRC metastasis. A) Transwell assays were performed to examine the potential migration of WT and GART‐OE CRC cells. Scale bar = 200 µm. B) Statistical results of the migration in WT and GART‐OE CRC cells are shown. C) Transwell assays were used to detect the potential migration of Ctrl and GART‐KD CRC cells. Scale bar = 200 µm. D) Statistical results of the migration in Ctrl and GART‐KD CRC cells are shown. E) Representative macroscopic appearances of the livers from nude mice with intrasplenic inoculation were shown. White arrowheads indicate the metastatic foci. F) Representative images of H&E staining of liver sections in nude mice. Scale bar = 200 µm. G,H) The relative mRNA expression of E‐cadherin, N‐cadherin and MMP‐9 in liver tissues from four groups, respectively. I,J) WB analysis of the expressions of GART and EMT markers in liver tissues. The data are expressed as the mean ± SD. *p<0.05, **p<0.01.

To assess the impact of GART on CRC development, transwell assays were then performed to observe cell metastasis in both WT and GART‐OE HCT‐116 and RKO cells. These experiments showed that GART could promote CRC cell migration (**Figure** **2**A,B). On the other hand, reducing the level of GART expression resulted in diminished CRC cell migration (Figure 2C,D).

We next established a liver metastasis model to further investigate the effects of GART on CRC metastasis in vivo. WT and GART‐OE HCT‐116 cells were respectively injected into the spleens of nude mice. Three days after injection of the cells into the spleen, the nude mice injected with WT HCT‐116 cells were intraperitoneally injected with DMSO and PEM, respectively (Figure S3, Supporting Information). Morphological observations and H&E staining demonstrated that an elevated level of GART led to a significant promotion of tumor metastasis to the liver, whereas treatment with PEM led to a clear reduction in hepatic metastasis (Figure 2E,F).

Epithelial–mesenchymal transition (EMT) is an important process of metastasis of malignant tumors, via which epithelial tumor cells lose their adhesion capability, which is necessary for obtaining mesenchymal cell mobility. RT‐qPCR and WB analyses both showed that the expression levels of GART were positively associated with multiple EMT molecules, namely N‐cadherin, vimentin and matrix metalloproteinase‐9 (MMP‐9) (Figure 2G–J). Interestingly, the administration of PEM caused a marked downmodulation in the expression of N‐cadherin and MMP‐9, whereas E‐cadherin was upregulated, indicating that PEM could reduce tumor metastasis to the liver (Figure 2G–J). Taken together, these data strongly suggest that GART is involved in enhancing the metastatic potential of CRC cells, and treatment with PEM can effectively reverse CRC cell migration.

### 2.3 GART Confers the Stemness in CRC

**Figure 3:**
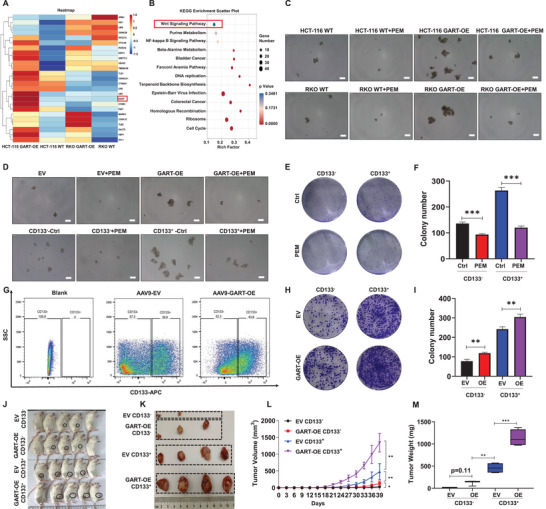
GART enhances the stemness in CRC. A) Heatmap of the differential target genes in WT and GART‐OE HCT‐116 cells. B) RNA sequencing (RNA‐seq) analysis highlighting the upregulated KEGG pathways. C) Representative images of formed spheroids derived from WT and GART‐OE cells treated with/without pemetrexed (PEM). Scale bar = 200 µm. D) Representative images of spheroids formed from primary mouse and human CRC cells treated with/without PEM. Scale bar = 200 µm. E) Colony formation assay of primary human CRC cells treated with PEM after CD133 sorting. F) The statistical analysis of the number of colonies in CD133^‐^ control (Ctrl) and PEM groups and CD133^+^ Ctrl and PEM groups. G) Flow cytometric analysis revealed sorting of AOM/DSS‐induced tumor cells with CD133 in EV and GART‐OE groups. H,I) Representative images of the colonies formed by CD133^‐^ and CD133^+^ EV and GART‐OE cells. J) Photographic images of xenograft mice captured at Day 39 (n = 6 in each group). K) Schematic images of xenografts from NOD/SCID mice. L) The time course of tumor growth in NOD/SCID mice. M) Average tumor weights of xenograft model mice. The data are expressed as the mean ± SD. *p<0.05; **p<0.01; ***p<0.001.

To identify the underlying molecular mechanism governing how GART is able to promote the proliferation and migration of CRC, RNA‐sequencing (RNA‐Seq) was performed to screen the differentially expressed genes between WT and GART‐OE HCT‐116 cells. Heatmap analysis showed *GART* was prominent gene among 25 genes with significant changes in the Wnt/β‐catenin signaling pathway in GART‐OE cells compared with WT cells (**Figure** **3**A). Kyoto Encyclopedia of Genes and Genomes (KEGG) enrichment analysis indicated that the Wnt/β‐catenin signaling pathway was involved in GART‐mediated CRC cell tumorigenicity (Figure 3B). Emerging evidence has indicated that Wnt/β‐catenin signaling pathway mediates the tumorigenicity and metastasis of numerous tumor types through regulating cell stemness.^[^ ^45^, ^46^, ^47^ ^]^ Based on the above analyses, the stemness mediated by GART in CRC cell lines and AOM/DSS‐induced primary tumor cells was examined using tumor sphere assay in vitro. The results showed that both the numbers and the diameters of the spheres derived from GART‐OE cells were increased compared with those derived from WT or empty vector (EV) cells, but PEM effectively led to the inhibition of sphere formation of the CRC cell lines and mouse primary tumor cells (Figure 3C,D; Figure S4, Supporting Information).

As CD133 is widely considered as a classic tumor stem cell marker,^[^ ^48^, ^49^ ^]^ we elected to culture primary tumor cells from patients with CRC in vitro, and sorted the CD133^‐^ and CD133^+^ cells for sphere and colony formation assays. Sphere‐forming experiments showed that the sphere formation capability of CD133^+^ cells was significantly higher compared with that of the CD133^‐^ cells; furthermore, PEM effectively inhibited the sphere formation capacity of CD133^+^ cells (Figure 3D; Figure S4D, Supporting Information). The colony formation assay also confirmed that CD133^+^ cells had a higher long‐term self‐renewal capability compared with CD133^‐^ cells, whereas PEM treatment led to a marked attenuation of the strong self‐renewal characteristics of the CD133^+^ cells (Figure 3E,F). Subsequently, flow cytometric analysis was performed to sort the CD133^+^ and CD133^‐^ cells from AOM/DSS‐induced CRC tumors in the EV and GART‐OE groups, respectively (Figure 3G). Colony formation assay demonstrated that the GART‐OE CD133^+^ cells exhibited a significantly higher level of self‐renewal activity compared with the EV CD133^+^ cells (Figure 3H,I). Furthermore, EV and GART‐OE CD133^+^ and CD133^‐^ cells respectively were subcutaneously injected into NOD/SCID mice (Figure S5, Supporting Information). These experiments confirmed that GART‐OE CD133^+^ cells generated tumors that were larger compared with those of the EV CD133^+^ cells, EV and GART‐OE CD133^‐^ cells; and the GART‐OE CD133^‐^ cells generated tumors that were larger than those of the EV CD133^‐^ cells, suggesting that GART could enhance the stemness of CRC (Figure 3J–M). Taken together, these findings suggest that GART confers stemness in CRC.

### 2.4 GART Mediates RuvB‐like AAA ATPase 1 (RUVBL1) to Promote CRC Malignancy and Metastasis

**Figure 4:**
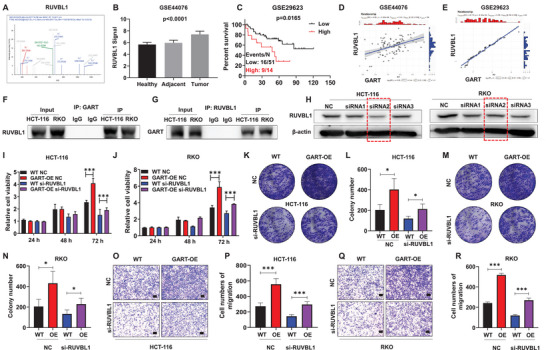
GART mediates RUVBL1 to promote CRC malignancy and metastasis. A) The secondary peak plot of RUVBL1 from mass spectrometry (MS) analysis. B) The mRNA levels of RUVBL1 in patients with CRC (n = 98) from the GSE44076 database were found to be higher than those in healthy (n = 50) or adjacent (n = 98) samples. C) Increased RUVBL1 was associated with poor CRC survival in the GSE29623 cohort. D,E) The association between *GART* and *RUVBL1* was analyzed using the CRC GSE44076 and GSE29623 databases. F,G) Co‐IP experiment indicated an interaction between GART and RUVBL1 in CRC cells. H) WB assay examined the knockdown efficiency of three siRNAs on HCT‐116 and RKO WT cells. I,J) CCK8 assay was used to detect the relative cell viability in WT and GART‐OE cells that were interfered with NC or RUVBL1 siRNAs. K–N) Colony formation assay examined the long‐term self‐renewal of WT and GART‐OE cells interfered with NC or RUVBL1 siRNAs. O–R) Transwell assay was used to determine the cell migration capabilities of WT and GART‐OE cells interfered with NC or RUVBL1 siRNAs. Scale bar = 200 µm. The data expressed as the mean ± SD. *p<0.05; ***p<0.001.

In order to unveil the potential mechanism underlying how GART facilitates CRC malignant progression, mass spectrometry (MS) followed by a co‐immunoprecipitation (Co‐IP) assay was performed to determine possible interaction partners of GART. The MS results revealed that hundreds of proteins might interact with GART. These proteins were further screened using KEGG analysis, mainly in the Wnt/β‐catenin signaling pathway and CRC GEO databases. This investigation revealed that RUVBL1 was closely associated with CRC development (**Figure** **4**A), and the expression levels of *RUVBL1* in the tumor and adjacent samples were increased compared with healthy samples in the GSE44076 database (Figure 4B). Moreover, the survival rate of CRC patients with high levels of *RUVBL1* was significantly decreased in the GSE29623 database (Figure 4C). In addition, by analyzing the GSE44076 and GSE29623 databases, *GART* expression was found to be positively correlated with *RUVBL1* (Figure 4D,E). The Co‐IP experiments also confirmed that *GART* could interact with *RUVBL1* in CRC cells (Figure 4F,G).

Subsequently, RUVBL1 expression was knocked down by using siRNAs to verify whether RUVBL1 exerted an important role in GART‐mediated CRC cell tumorigenicity and migration. Three siRNAs were designed for interfering with RUVBL1. WB assay showed that siRNA2, the second synthetic siRNA, was associated with a satisfactory knockdown efficiency in WT HCT‐116 and RKO cells (Figure 4H). Consequently, siRNA2 was used for the subsequent experiments. CCK8 and colony formation assays showed that GART‐OE cells had a relatively higher self‐renewal capacity, whereas knocking down RUVBL1 in GART‐OE cells led to a significant decrease in the self‐renewal rate of CRC cells (Figure 4I–N; Figure S6, Supporting Information). Furthermore, transwell assay revealed that GART‐OE cells had a strong migration capability, although the migration trend was inhibited by siRUVBL1 (Figure 4O–R). Collectively, these data demonstrate that RUVBL1 participates in GART‐mediated CRC cell tumorigenicity and migration.

### 2.5 GART Methylates the K7 Site of RUVBL1 to Inhibit the Ubiquitination of RUVBL1

**Figure 5:**
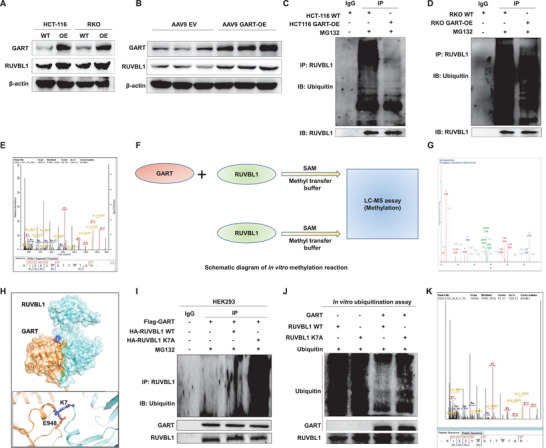
GART methylates the K7 site of RUVBL1 to inhibit the ubiquitination of RUVBL1. A,B) WB was used to detect the expression levels of GART and RUVBL1 in WT and GART‐OE CRC cells, and AOM/DSS‐induced CRC tumor tissues, respectively. C,D) The ubiquitination of RUVBL1 in WT and GART‐OE CRC cells was shown. E) LC‐MS assay showed the methylation of the E5, K7, and K11 sites of RUVBL1 in GART‐OE HCT‐116 cells. F) Schematic diagram of GART acting as a novel methyltransferase. G) The secondary peak plot of methylation at RUVBL1 K7, as indicated by LC‐MS. H) The molecular pattern diagram of the interaction between GART and RUVBL1. I) WB was performed to detect the ubiquitination of RUVBL1 upon transfection of GART with flag tag and RUVBL1 WT or K7A with HA tag in HEK293 cells, respectively, under the condition of the treatment of MG132. J) in vitro ubiquitination assay revealed that GART significantly inhibited RUVBL1 ubiquitination. K) The secondary peak plot of methylation at the RUVBL1 K7 site between GART WT and GART E948A groups, as detected by LC‐MS.

Our experiments continued to clarify the mechanism through which GART could mediate RUVBL1 to regulate the Wnt/β‐catenin signaling axis. WB analysis confirmed that the levels of both the GART and RUVBL1 proteins were elevated in both GART‐OE CRC cell lines and the AOM/DSS‐induced tumor tissues (**Figure** **5**A,B). The ubiquitin–proteasome system has been shown to mediate the degradation of a wide range of proteins, whereas inhibition of protein ubiquitination effectively maintains protein stability.^[^ ^50^, ^51^ ^]^ Therefore, it was possible to surmise that upregulation of RUVBL1 protein could result as a consequence of GART impeding the ubiquitination of RUVBL1. To investigate this possibility, the levels of RUVBL1 ubiquitination in WT and GART‐OE HCT‐116 and RKO cells were compared, respectively. WT and GART‐OE cells were treated with MG132, a proteasome inhibitor, at a concentration of 20 µM for 6 h prior to harvesting the cells. Co‐IP experiments demonstrated that overexpressed GART led to less ubiquitination and degradation of RUVBL1 compared with WT cells (Figure 5C,D).

As GART is a formyltransferase, we initially considered that GART could induce RUVBL1 formylation to affect its ubiquitinated degradation. To explore this hypothesis, liquid chromatography−mass spectrometry (LC‐MS) was performed to detect the RUVBL1 formylation levels in WT and GART‐OE HCT‐116 cells; however, no formylation of RUVBL1 protein was identified in these cells. Surprisingly, the methylation of E5, K7, and K11 sites of RUVBL1 in GART‐OE cells was detected (Figure 5E). To further confirm whether GART possessed methyltransferase activity, in vitro methylation experiments were performed (Figure 5F), which indicated that GART could methylate the RUVBL1 K7 site (Figure 5G). Moreover, the molecular pattern diagram showed that GART interacted with the K7 site of RUVBL1 (Figure 5H). In addition, the amino acid lysine‐7 of RUVBL1 was mutated into alanine (K7A), and GART was subsequently co‐transfected with WT RUVBL1 or RUVBL1 K7A mutant, respectively, in HEK293 cells. Ubiquitination assay indicated that the K7 site mutation of RUVBL1 effectively reversed the low ubiquitination level of RUVBL1 that was mediated by GART (Figure 5I). Ubiquitination experiments in vitro were also used to further confirm that GART could inhibit K7 site ubiquitination through inducing methylation at the K7 site and stabilizing RUVBL1 (Figure 5J). Based on the molecular docking simulations via computer technology, the K7 site of RUVBL1 binds to the E948 site of GART (Figure 5H). The E948 site is a key amino acid site in the active pocket of GART, which can be targeted by PEM.^[^ ^52^ ^]^ Our hypothesis was that the E948 site of GART could be a methyltransferase active site. The in vitro methylation experiments and LC‐MS detection method verified the methylation of the RUVBL1 K7 site (Figure 5K), which was consistent with the above results. The methylation modification of RUVBL1 K7 site was undetectable in an experiment where the E948 site of GART was mutated to alanine (E948A). Taken together, these experiments have shown that the E948 site of GART binds to the K7 site of RUVBL1, leading to the methylation of the K7 site and decreased ubiquitination of RUVBL1.

### 2.6 GART Contributes to CRC Progression via the RUVBL1‐Mediated β‐Catenin Signaling Pathway

**Figure 6:**
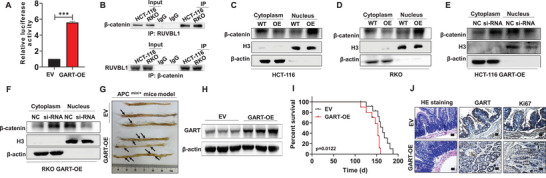
GART mediates the RUVBL1/β‐catenin signaling axis to promote CRC growth. A) Dual‐luciferase reporter assay was used to determine the level of activation of the Wnt/β‐catenin signaling pathway in HEK293 cells. B) Co‐IP assay confirmed that RUVBL1 physically interacted with β‐catenin in HCT‐116 and RKO cells. C–F) WB assays detected the nuclear and cytoplasmic distribution of β‐catenin in WT and GART‐OE CRC cells, and GART‐OE CRC cells interfered with NC and siRUVBL1, respectively. G) Macroscopic appearance of tumors in the large intestine of APC^min/+^ mice in the EV and GART‐OE groups was shown. H) WB analysis of GART expression in the large intestine samples of APC^min/+^ mice in the EV and GART‐OE groups. I) Kaplan‐Meier survival curve analysis of the APC^min/+^ mice in the EV and GART‐OE groups (n = 10 in each group). J) Representative images of H&E and immunohistochemistry (IHC) staining of GART and Ki67 in the APC^min/+^ mice tumor sections. Scale bar = 50 µm. The data are expressed as the mean ± SD. ***p<0.001.

It is known that the RUVBL1‐mediated Wnt/β‐catenin signaling axis has an important role in the occurrence and development of numerous types of malignancies.^[^ ^53^, ^54^ ^]^ Our aim was to identify whether GART could mediate the RUVBL1/catenin signaling axis in the development of CRC. To meet this end, dual‐luciferase reporter experiments were performed, which revealed that overexpression of GART definitively activated the Wnt/β‐catenin signaling pathway (**Figure** **6**A). IHC staining showed that RUVBL1 and β‐catenin were significantly increased in CRC patients compared with healthy controls (Figure S7, Supporting Information). Co‐IP assay was then used to validate the interaction of RUVBL1 with β‐catenin in HCT‐116 and RKO cells (Figure 6B). IF assays confirmed the co‐localization of GART and RUVBL1 in CRC patient tissues, as well as the co‐localization of RUVBL1 and β‐catenin in both cytoplasm and nucleus (Figure S8 and S9, Supporting Information). Furthermore, WB analysis proved that a high expression level of GART could promote the nuclear translocation of β‐catenin (Figure 6C,D). Subsequently, GART‐OE HCT‐116 and RKO cells were respectively transduced with siRUVBL1. β‐catenin was found to be clustered in large quantities in the cytoplasm relative to the nucleus in siRUVBL1 cells compared with NC cells, suggesting that restricting RUVBL1 expression led to an inhibition of β‐catenin signal transduction (Figure 6E,F).

*Adenomatous polyposis coli* (*APC*), a classic tumor suppressor gene, has been shown to negatively regulate the Wnt signaling pathway via inhibiting β‐catenin expression.^[^ ^55^, ^56^ ^]^ APC^min/+^ mice have been widely studied and used as a spontaneous model of CRC.^[^ ^57^ ^]^ Morphological observations revealed that an increased level of GART led to marked acceleration of tumor growth in the colorectum of APC^min/+^ mice (Figure 6G; Figure S10, Supporting Information). WB assay revealed that the expression level of GART was significantly increased in the colorectal samples from GART‐OE group compared with EV group (Figure 6H). Moreover, Kaplan‐Meier survival curve analysis showed that the mice in GART‐OE group had markedly shorter survival times compared with mice in EV group (Figure 6I). Furthermore, IHC and H&E staining experiments verified that the level of GART was clearly increased in the intestines of the GART‐OE mice compared with the EV mice (Figure 6J). Taken together, these findings suggest that GART can augment CRC progression via the RUVBL1‐mediated Wnt/β‐catenin signaling pathway both in vitro and in vivo.

### 2.7 Treatment with PEM Effectively Suppresses Tumor Growth in a PDX Mouse Model and Patients with CRC

**Figure 7:**
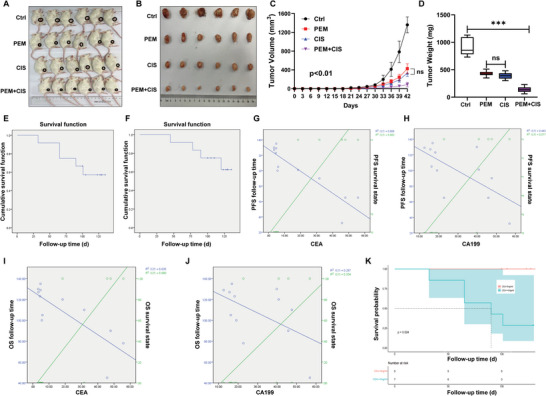
Treatment with pemetrexed effectively suppresses tumor growth in a PDX mouse model and patients with CRC. A) Photographic images of xenografts of CRC patient‐derived xenograft (PDX)‐modeled mice treated with pemetrexed (PEM) and cisplatin (CIS), individually or combined, for 42 days. B) Schematic images of PDX tumors from NOD/SCID mice in control (Ctrl), PEM, CIS and PEM+CIS groups are shown. C) Tumor growth curve of the PDX mice in Ctrl, PEM, CIS and PEM+CIS groups . D) The mean PDX tumor weight of the PDX mice in the Ctrl, PEM, CIS, and PEM+CIS groups. E,F) With a combined treatment regimen, the median progression‐free survival (mPFS) and median overall survival (mOS) of patients with CRC reached 107.7 days and 117.1 days, respectively. G,H) The effect of PEM treatment on PFS of patients with CRC was negatively correlated with CEA or CA199 levels I,J) The effect of PEM treatment on OS of patients with CRC was found to be negatively associated with the CEA or CA199 level. The data are expressed as the mean ± SD. ***p<0.001.

To evaluate the antitumor efficiency of PEM, a selective inhibitor of GART, at the clinical level, PEM was administered to PDX mice. Both PEM and cisplatin (CIS) were found to effectively restrain tumor growth in PDX mice, and the combination of PEM and CIS exerted an even more pronounced inhibitory effect on tumor growth (**Figure** **7**A–D).

In addition, a series of clinical trials of PEM for CRC treatment were carried out. The screening, treatment, evaluation and statistical methods of patients with CRC in clinical trials for this study are detailed in Additional file 1. The baseline characteristics of the assessed patients are shown in **Table** **2**. Of the eight surviving patients with CRC, five were treated with PEM in combination with capecitabine, and three were treated with PEM in combination with tegafur. The median progression‐free survival (mPFS) rate was 107.7 days (95% confidence interval: 87.75‐127.78 days), and the median overall survival (mOS) rate was 117.1 days (95% confidence interval: 100.78‐133.47 days) in patients receiving the combined treatment, suggesting that PEM might help to prolong the survival of patients (Figure 7E,F).

**Table 2:** The baseline characteristics of the assessed CRC patients

Characteristics	Number	Mean	SD	HR [95%Cl]		χ^2^	*P*
Gender						2.244	0.134
Female	4	82	14.693	53.2	110.8		
Male	8	117.5	10.717	96.5	138.5		
Age year							
<65	4	103.25	20.568	62.937	143.563	0.392	0.531
≥65	8	106.563	10.511	85.96	127.165		
Primary tumor site							
Left	9	103	12.774	77.962	128.038	0.307	0.579
Right	3	118.333	7.076	104.464	132.203		
ECOG							
1	11	111.649	10.373	91.319	131.98	2.398	0.121
2	1	65	0	65	65		
CEA							
<5 ng/mL	5						
≥5 ng/mL	7						
CA199							
<37 U/mL	7	118.571	10.138	98.702	138.441	1.063	0.303
≥37 U/mL	5	91.6	17.085	58.114	125.086		
Differentiation							
Well‐differentiatied	9	122.222	8.207	106.137	138.308	6.722	0.01
Poor‐differentiated	3	66	19.925	26.947	105.053		

Intriguingly, 25% of the patients were in partial remission (PR), 33.3% of the patients were in stable disease (SD), and 41.7% of the patients were in the progressive disease (PD) phase. The disease control rate (DCR) was 25%, and the objective response rate (ORR) reached 58.3%. This analysis also revealed that PEM efficacy was negatively associated with the vital biomarkers for CRC patient outcomes, such as the levels of carcinoembryonic antigen (CEA) and carbohydrate antigen199 (CA199) (Figure 7G–J). Kaplan‐Meier survival curve analysis indicated that the PFS rate of CRC patients with a low CEA level was significantly higher compared with patients with a high CEA level following PEM treatment (Figure 7K), suggesting that the treatment with PEM could alleviate the progression of CRC clinically.

## 3 Discussion

Cancer stemness is responsible for both the occurrence and incurability of malignant CRC.^[^ ^58^, ^59^ ^]^ A wide range of studies have demonstrated that CCSCs fulfill an essential role in the tumor initiation, maintenance, metastasis and chemoresistance of CRC.^[^ ^60^, ^61^, ^62^, ^63^ ^]^ CD133 is one of the most representative cell‐surface markers of cancer stem cells, which has been demonstrated by an increasing number of studies on liver cancer, lung cancer, breast cancer, pancreatic ductal adenocarcinoma, and other solid cancers.^[^ ^64^, ^65^, ^66^, ^67^ ^]^ For example, CD133^+^ tumor‐initiating cells have been shown to drive intra‐tumoral heterogeneity in glioblastoma.^[^ ^68^ ^]^ CD133 exhibits both high expression at the protein level and strong tumorigenic capability in CRC cells.^[^ ^69^ ^]^ CD133 has also been demonstrated to be involved in Wnt/β‐catenin signaling pathway‐mediated CRC cell self‐renewal, metastasis and poor outcome in patients,^[^ ^70^, ^71^, ^72^ ^]^ and the Wnt/β‐catenin signaling pathway promotes the self‐renewal and metastasis of numerous types of tumors through regulating tumor cell stemness. In addition, CD133 has been shown to be closely associated with CRC cell invasion, metastasis and patient prognosis.^[^ ^73^, ^74^, ^75^ ^]^ Consistently with these studies, the results from the present study have verified that CD133^+^ CRC primary cells possess high degrees of stemness and tumorigenicity. Therefore, directly targeting CD133 may be an effective method to eliminate cancer stem cells. However, current therapeutic strategies targeting CD133, such as those incorporating immunotoxins, T‐cell therapy, aptamers and antibody‐conjugated nanoparticles, are less than optimal due to CD133 splice variants and aberrant post‐translation modification.^[^ ^76^, ^77^, ^78^, ^79^, ^80^ ^]^ The glycosylated epitope of CD133 is easily mutated during the development of malignant tumors, and its expression is also susceptible to changes in oxygen concentration.^[^ ^81^, ^82^, ^83^ ^]^ In the face of malignant characteristics of CRC, such as refractory disease and the ease with which patients relapsed, the present study has demonstrated that a novel methyltransferase GART is involved in the malignant development of CRC via RUVBL1/β‐catenin‐mediated cancer stemness.

GART, as a key folate synthase in the purine *de novo* synthesis pathway, has three enzyme activities: glycinamideribonucleotide formyltransferase, aminoimi‐dazole ribonucleotide synthase, and glycinamide ribonucleotide synthase.^[^ ^84^, ^85^ ^]^ High expression levels of GART are associated with poor prognosis in patients with hepatocellular carcinoma (HCC), and they promote the tumorigenicity of HCC through regulating cyclins.^[^ ^41^ ^]^ A recent study also showed that vestigial‐like family member 3 upregulates GART to enhance the activity of *de novo* nucleotide synthesis, leading to the malignancy of lung and breast cancer cells.^[^ ^86^ ^]^ However, the role and mechanism of GART in CRC progression remains unclear. It is worth noting that GART is highly expressed in the intestinal lamina propria in Crohn's disease, and affects the rate of apoptosis during development of the disease.^[^ ^87^ ^]^ Patients with Crohn's disease have an increased risk of CRC diagnosis and CRC mortality.^[^ ^88^ ^]^ In the present study, the high expression level of GART in patients with CRC was first validated from GEO databases, and these were found to be significantly elevated in CRC samples compared with the healthy and adjacent tissues. Moreover, Kaplan‐Meier analysis demonstrated that CRC patients with elevated GART were associated with poor survival rates. However, it remained unclear whether GART could promote the malignant progression of CRC via modulating tumor stemness. In this study, CRC cell lines, mouse models and human tumor tissue were all used to reveal a novel role of GART in CRC, especially in CD133^+^ CRC stem cells.

Intriguingly, the data obtained from high‐throughput RNA‐seq, the luciferase reporter assay and nucleoplasmic protein isolation experiments, and the APC^min/+^ spontaneous CRC mouse model confirmed that the Wnt/β‐catenin signaling pathway is involved in GART‐mediated CRC cell self‐renewal. Proteins that could interact with GART were further screened by MS, and the downstream protein RUVBL1 was identified. It has been reported that circMYO10 promotes osteosarcoma progression via the miR‐370‐3p/RUVBL1/β‐catenin/LEF1 complex axis.^[^ ^53^ ^]^ The present study also showed that an elevated level of GART induced a high level of expression of RUVBL1 in CRC cells and tissues. To explore the potential underlying mechanism, LC‐MS was performed to detect the presence of formylation modification catalyzed by GART; however, no formylation in RUVBL1 was detected. Of greater interest was that fact that these experiments revealed, to the best of our knowledge for the first time, that methylation modifications of RUVBL1 existed in GART‐OE CRC cells. Methylation is a common and critical form of protein post‐translational modification that regulates protein function and tumor development.^[^ ^89^, ^90^, ^91^, ^92^ ^]^ Krushkal et al.^[^ ^93^ ^]^ used Transcriptional Pharmacology Workbench (the bioinformatics tool) to identify that, in NCI‐60 cancer cells upon treatment with five antitumor agents, the transcriptional changes of GART and five other genes influenced DNA methylation and demethylation. To date, there is insufficient evidence to conclude decisively that GART has methylase activity. In the present study, GART was identified to promote methylation at the RUVBL1 K7 site via in vitro methylation experiments, which strongly supported a novel methyltransferase function of GART. It is known that methylation of lysine regulates protein stability through the ubiquitin–proteasome degradation pathway.^[^ ^94^ ^]^ We were able to further confirm that GART‐dependent methylation of the RUVBL1 K7 site promoted the stable expression of RUVBL1 by utilizing the methods of lysine‐targeted mutation technology and in vitro methyl‐ubiquitination assay.

Based on the molecular docking simulations via computer technology, we found that, as a novel methyltransferase, the enzymatic activity of GART was centered on E948, which could mediate methylation of the RUVBL1 K7 site. Golani et al.^[^ ^52^ ^]^ demonstrated that PEM targeted active pockets composed of amino acids such as R871 and R897 of GART to achieve anti‐tumor effects. Coincidentally, through protein structure analysis, we found that the E948 site was the key site in the pocket where PEM targets GART. Thus, we were able to hypothesize that PEM might be used to treat CRC by targeting the E948 site of GART to inhibit its methyltransferase activity, so as to suppress the RUVBL1‐mediated β‐catenin signaling pathway. Although our study revealed that RUVBL1 bound to the E948 site of GART, but which site of PEM interacting with GART still need further exploration. In general, PEM is clinically used in combination with other chemotherapy drugs, such as CIS, for  improved antitumor activity,^[^ ^95^ ^]^ although whether it could achieve a therapeutic effect by intervening in tumor stemness requires scientific confirmation. Previous studies on PEM targeting cancer stem cells (CSCs) to solve the puzzle of tumor relapse have been reported in the case of lung cancer,^[^ ^96^, ^97^ ^]^ but are rare in cases of CRC. In the present study, a series of spherical experiments were designed, demonstrating that PEM could intervene in the stemness of CRC in a variety of models. A combination of PEM and other drugs was also adopted to gain satisfactory effects and broad treatment prospects for curing CRC in both a PDX mouse model and in clinical patients, respectively. Our clinical application of PEM in combination with capecitabine or tegafur effectively alleviated the progression of CRC, which was consistent with the findings of another research group.^[^ ^98^ ^]^

In conclusion, in the present study we have characterized the role of GART in promoting CRC cell tumorigenicity and invasion via enhancing cancer stemness; furthermore, we have revealed the underlying mechanism through which GART activates Wnt/β‐catenin signaling, by methylating RUVBL1 to inhibit its ubiquitination degradation. More importantly, clinical regimens in combination with PEM may provide promising theoretical support and be of therapeutic value in terms of the treatment of CRC in the future.

## 4 Experimental Section

### Gene Expression Profiling

Gene expression profiling (GEP) cohorts were acquired from the Gene Ontology (GEO) databases as described previously.^[^ ^99^ ^]^ The GSE44076 cohort comprised 98 paired normal adjacent mucosa and tumor tissues and 50 healthy colon mucosae (https://www.ncbi.nlm.nih.gov/geo/query/acc.cgi?acc = GSE44076), whereas the GSE29623 cohort comprised primary tumor specimens from 65 patients (40 males and 25 females) with colon cancer.^[^ ^100^ ^]^

### Antibodies and Reagents

The antibodies used in the present study were as follows: anti‐GART (cat. no. 13 659‐1‐AP, ProteinTech Group, China); anti‐RUVBL1 (cat. no. 10 210‐2‐AP, ProteinTech Group, China); anti‐β‐catenin (cat. no. 51 067‐1‐AP, ProteinTech Group, China); anti‐β‐actin (cat. no. 4970S, Cell Signaling Technology, USA); anti‐ubiquitin (cat. no. 10 201‐2‐AP, ProteinTech Group, China); anti‐E‐cadherin (cat. no. 20 874‐1‐AP, ProteinTech Group, China); anti‐N‐cadherin (cat. no. 22 018‐1‐AP, ProteinTech Group, China); anti‐vimentin (cat. no. 10 366‐1‐AP, ProteinTech Group, China); and anti‐histone‐H3 (cat. no. 17 168‐1‐AP, ProteinTech Group, China). All the primary antibodies were diluted in QuickBlock™ primary antibody dilution buffer for western blot (cat. no. P0256, Beyotime Biotechnology, China).

Tetracycline (TET) was obtained from Yeasen Biotechnology (Shanghai) Co., Ltd (cat. no. 60212ES25, Shanghai, China). Rabbit IgG (cat. no. a7016) was purchased from Beyotime Institute of Biotechnology (Shanghai, China). Puromycin was obtained from Merck KgaA (Darmstadt, Germany), and the Cell Counting Kit 8 (CCK8) assay was purchased from SinoMol Biotechnology (cat. no. CCK‐810, Nanjing, China).

### Cell Lines and Cell Culture

The human CRC cell lines HCT‐116 and RKO were cultured in RPMI‐1640 medium (Biological Industries, Israel). HEK293 cells were cultured in DMEM (Thermo Fisher Scientific, Inc., USA). The culture medium was supplemented with 10% Gibco^®^ fetal bovine serum (Thermo Fisher Scientific, Inc., USA), 100 U ml^−1^ HyClone™ penicillin (Thermo Fisher Scientific, Inc., USA) and 100 µg ml^−1^ HyClone™ streptomycin (Thermo Fisher Scientific, Inc., USA). All cells were cultured at 37 °C in a 5% CO_2_ incubator.

### Plasmids and Transfection Studies

Plasmids containing human GART cDNA and GART shRNA cassettes were purchased from Hunan Fenghui Biotechnology Co., Ltd. (Changsha, China). GART coding sequence was cloned into a pCDH vector with Flag tags (Hunan Fenghui Biotechnology Co., Ltd, China); GART‐targeting shRNA was inserted into a pLKO vector (Hunan Fenghui Biotechnology Co., Ltd, China), wherein a TET‐inducible promoter acted as the operator. The packaging vector (containing VSVG, PLP1, and PLP2) and expression vector were co‐transfected into HEK293 cells for obtaining lentivirus via using Lipofectamine Transfection Reagent (cat. no. 40802ES03, Yeasen Biotechnology (Shanghai) Co., Ltd., China). After 48 h, the virus supernatant was collected, and stored at −80 °C. The transfected CRC cells were screened by puromycin with high transduction efficiency.

### Transient Transfection

Small interfering RNA (siRNA) was purchased from Shanghai GenePharma Co., Ltd. (Shanghai, China). The base sequences used to silence RUVBL1 were as follows: sense strand (5′→3′): CCAUUGGGCUGCGAAUAAATTdTdT; and antisense strand (5′→3′): UUUAUUCGCAGCCCAAUGGTTdTdT. When the HCT‐116 or RKO cells had attained 70% confluency, they were transfected with 100 nM si‐RUVBL1 or negative control (NC) siRNA using Lipofectamine Transfection Reagent. After 6 h, normal Complete™ medium was used for culture of the transfected cells at 37 °C. After 48 h, the cells were used for the subsequent experiments.

### Cell Proliferation and Colony Formation Assays

The proliferative capability and viability of the cells at 24, 48, and 72 h were detected using the CCK‐8 assay method. The number of viable cells were measured by microplate reader at an absorbance of 450 nm.

For colony formation assay, cells (1 × 10^3^ cells per well) assigned to different treatment groups were cultured in 6‐well plates. At the termination of the experiments, the colonies were washed with PBS, fixed with 4% paraformaldehyde (cat. no. BL539A, Biosharp, China) for 10 min, and stained with 0.2% crystal violet (cat. no. C0121, Beyotime Biotechnology, China) for 30 min. Over 50 cells were counted in the colony.

### Tumorsphere Formation Assay

CRC cells (1 × 10^4^ cells per well) were seeded in 6‐well plates with ultra‐low attachment in sphere formation medium. Subsequently, CRC cells were cultured in a cell incubator to form tumorspheres at 37 °C. After 1‐2 weeks, the images of CRC tumorspheres were captured by inverted fluorescence microscope (Optika IM‐3FL4, Italy) at a magnification of ×100.

### Transwell Assay

Cell migration was determined by transwell assay. CRC cells (1 × 10^5^) were seeded into 8‐µm‐pore upper chambers in serum‐free RPMI‐1640 medium serum, and then incubated with RPMI‐1640 medium containing 10% FBS in the lower chambers of 12‐well plates. After 24‐48 h, cells were washed with PBS, fixed with 4% paraformaldehyde, and subsequently stained with crystal violet. Images of the migrated cells were then captured under inverted fluorescence microscope.

### Western Blotting (WB) and Co‐Immunoprecipitation (Co‐IP) Assays

WB assay was performed as described previously.^[^ ^101^ ^]^ Co‐IP assay was performed using a Pierce Direct Magnetic IP/Co‐IP kit (Thermo Scientific. Inc.) also according to the manufacturer's instructions.

### RNA Sequencing (RNA‐Seq) Assays

The total RNA from cell samples was extracted by using TRIeasy (cat. no.10606ES60, Yeasen Biotechnology, Shanghai,) according to the protocol provided by the manufacturer. The integrity, quantity, and purity of total RNA were analyzed by Bioanalyzer 2100 and RNA 6000 Nano LabChip Kit (Agilent, Palo Alto, CA, USA). Polya (polyadenylated) bearing mRNAs were specifically captured through two rounds of purification by oligo magnetic beads (Dynabeads Oligo (dT), cat. no.25‐61005, Thermo Fisher, USA). The captured mRNA was fragmented using a magnesium ion fragmentation kit (NEBNextR Magnesium RNA Fragmentation Module, cat. no. E6150S, USA) under high‐temperature conditions at 94 °C for 5–7 min. The cleaved RNA fragments were reverse‐transcribed to create the final cDNA library using the mRNA‐Seq sample preparation kit (Illumina, San Diego, CA, USA). Next, the paired‐end sequencing was performed on an Illumina Novaseq™ 6000 (LC Bio Technology CO., Ltd. Hangzhou, China) according to the vendor's recommended protocol. KEGG was employed to screen the differential genes by comparing clean data of WT & GART‐OE cells.

### 4.1 Label Free Mass Spectrometry (MS) Analysis

SDS‐PAGE was used for protein extraction, and gel bands were digested with sequencing‐grade trypsin (Promega, USA). The extract peptide segments after enzymolysis were analyzed by the timsTOF Pro 2 (Bruker Daltonics) mass spectrometry. Fragment spectra were analyzed by MaxQuant search engine (version 1.6.15.0). The conservative motifs matching the target protein were searched in the European Bioinformatics Institute (EBI) database by InterProScan tool. GO enrichment analysis was used to annotate the functional information of the target protein sequence. The ANNEX software was run to further supplement the annotation information and establish links between different GO categories. The KEGG pathway database was used to analyze representative pathways for differentially expressed proteins.

### Reverse Transcription‐Quantitative PCR (RT‐qPCR)

Sequences of the primers are presented in **Table** **3**. Total RNA was extracted from CRC tissues by using TRIzol™ reagent [Yeasen Biotechnology (Shanghai) Co., Ltd., Shanghai, China] in accordance with the manufacturer's instructions. The cDNA was generated using the reverse transcription kit (Vazyme Biotech Co., Ltd., Nanjing, China). The qPCR test was performed using SYBR^®^ mix (Vazyme Biotech Co., Ltd.) using GAPDH for normalization. Analytikjena qPCR soft 4.0 (Germany) was used to run qPCR reaction program as follows: predenaturation temperature was 95 °C, 3 min; denaturation temperature was 95 °C, 10 s; annealing temperature was 60 °C, 59 s; a total of 40 cycles. The 2^−ΔΔCt^ method was used to calculate the relative expression levels of target genes.

**Table 3:** Sequences of the primers used in the study

Targets	Sequences
E‐cadherin‐F	GCCCTGCCAATCCCGATGAAA
E‐cadherin‐R	GGGGTCAGTATCAGCCGCT
MMP9‐F	CAGAGATGCGTGGAGAGT
MMP9‐R	TCTTCCGAGTAGTTTTGG
N‐cadherin‐F	AGCCAACCTTAACTGAGGAGT
N‐cadherin‐R	GGCAAGTTGATTGGAGGGATG
GAPDH‐F	GTCTCCTCTGACTTCAACAGCG
GAPDH‐R	ACCACCCTGTTGCTGTAGCCAA

### Immunohistochemistry (IHC)

IHC staining for paraffin‐embedded tissue sections of human and mouse normal and tumor specimens was performed in line with standard protocols. Specimens were deparaffinized, rehydrated under antigen retrieval and blocked with hydrogen peroxide and goat serum, followed by incubation with the corresponding primary antibody overnight at 4 °C. The next day, the secondary antibody was applied, and the tissue sections were incubated with the antibody for 45 min at 37 °C, followed by treatment with SABC Reagent solution (cat. no. SA1021 & SA1022, BOSTER Biological Technology co.Ltd, China) for 30 min at 37 °C. The sections were stained by incubation with 3,3′‐diaminobenzidine (DAB). Light counterstaining was performed with hematoxylin, and finally images were captured under the inverted fluorescence microscope.

### Establishment of the Subcutaneous Xenograft Model

WT and GART‐OE HCT‐116 cells (1 × 10^6^) were subcutaneously injected into the bilateral flanks of 6–8‐week‐old nude mice. The tumor growth was monitored every 2 days, and tumor volume was calculated according to the formula: length × width^2^ /2. Tumors were harvested for photographing, and were weighed once they had reached a diameter of 15 mm.

### Establishment of the Liver Metastasis Model

The liver metastasis model was established via intrasplenic inoculation f CRC cells. WT and GART‐OE HCT‐116 cells (1 × 10^6^) were injected into the spleens of 4‐week‐old nude mice, respectively. After 7 weeks, the nude mice were sacrificed by cervical dislocation under anesthesia with 1% sodium pentobarbital, and the livers were harvested and photographed. Finally, the samples were fixed in 4% paraformaldehyde solution for further H&E staining.

### The Azoxymethane (AOM)/Dextran Sulfate Sodium (DSS)‐Induced CRC Model

Empty vector (EV) and AAV9‐loaded GART‐OE vector were purchased from GeneChem, Inc. Tail vein injections were carried out as follows: 1 × 10^11^ virus genome (vg) of AAV9 in 100 µL of saline was injected into the tail veins of 4‐week‐old BALB/c and APC^min/+^ mice. The transduction efficiency of AAV9 was evaluated using WB assay.

The AOM/DSS‐induced CRC model was manipulated after AAV9 transduction for 2 weeks. Briefly, azoxymethane AOM (Sigma‐Aldrich) was intraperitoneally injected into BALB/c mice at a dose of 10 mg kg^−1^ body weight. The next day, the mice were fed with 2.5% DSS (MP Biomedicals LLC, Irvine, CA, USA) dissolved in distilled water for 1 week, followed by normal drinking for 2 weeks. The procedure was repeated three times. Finally, the mice were sacrificed for further histological and molecular biological investigations.

### Establishment of the APC^min/+^ mouse‐induced CRC model

The breeding colony was established by crossing heterozygous male C57BL/6J‐APC^min/+^ mice with WT female C57BL/6J mice. Genomic DNA was prepared from the tail biopsies. Multiplex PCR was used for genotype identification of the offspring. The oligonucleotides used has the following sequences: sense, 5′‐ATACTACGGTATTGCCCAGC‐3′; and antisense, 5′‐TGTTGTTGGATGGTAAGCAC‐3′. The expected size of the PCR product was 122 bp for the WT animals. Two additional bands of 122 and 159 bp were obtained from the C57BL/6J‐APC^min/+^ mice.

EV and AAV9‐carried GART‐OE vectors were purchased from GeneChem, Inc. The tail vein injections were implemented as follows: 1 × 10^11^ vg of AAV9 in 100 µL of saline solution was injected into the tail veins of 4‐week‐old APC^min/+^ mice. Finally, the animals were sacrificed for further histomorphological and molecular biology experiments.

### Establishment of the CRC Patient‐Derived Tumor Xenograft (PDX) Model

The PDX was generated by using the surgically removed tumor tissue from a patient with CRC at the Department of Proctology, Nanjing Hospital of Chinese Medicine affiliated to Nanjing University of Chinese Medicine. The tumor slices were transplanted subcutaneously into 6‐week‐old NOD/SCID mice (n = 6 mice in each group) under anesthesia with 1% sodium pentobarbital. The tumors were collected once their sizes reached 500 mm^3^, and subsequently the tumor tissues were divided into 2.5 × 2.5 × 2.5 mm^3^ pieces and subcutaneously implanted into the NOD/SCID mice again. This process was then repeated three times, and once the tumor size had reached 100‐150 mm^3^, the mice were randomly divided into the control (Ctrl), PEM administration, cisplatin (CIS) administration and PEM and CIS combination groups.

All the animal experiments in this study were performed in accordance with the recommendations of the Care and Use of Laboratory Animals, and the Guidelines of Institutional Ethics Review Boards of Nanjing University of Chinese Medicine (Nos. 202202A014, 202111A038, 202203A009, 202205A053, and 202301A004).

### PEM Treatment Study in CRC Patients

PEM treatment study in CRC patients were performed by Dr. Xing Cui from the Second Affiliated Hospital of Shandong University of Traditional Chinese Medicine. The study was in accordance with the Declaration of Helsinki 2013, and approved by the institutional review board of the Second Affiliated Hospital of Shandong University of Traditional Chinese Medicine (KY‐001). Informed consent was obtained from all patients.

### Molecular Docking Method

Crystal Structure of GART (PDB ID: 7JG0) and RUVBL1 (PDB ID: 2C9O) were retrieved from PDB bank (https://www.rcsb.org). The missing residues (1‐8) of RUVBL1 structure were built by using the Molecular Operating Environment (MOE) software and were subsequently refined by molecular dynamics (MD) simulations for 50 ns. The MD simulations were performed by the Desmond protocol module in Schrodinger that adopted the OPLS3 force field. To prevent solvent molecules from all in a muddle, the orthorhombic periodic boundary condition was introduced. The sampling was carried out under the temperature of 300 K and constant pressure consumption. Both structures were then prepared by Schrödinger software using the “protein preparation” with default settings. Finally, “Protein‐Protein Docking” module of Schrödinger software was used to predict the interactions between RUVBL1 and GART. “Standard” mode was used and other settings were as default values.

### Statistical Analysis

GraphPad Prism Software (version 8.0.1) was used for statistical analysis. All results were presented as the mean ± SD. All comparisons between 2 groups were performed using two‐tailed unpaired Student's t‐test for the condition of homogeneity of variance (*p*>0.1). Otherwise, unpaired t‐test with Welch's correction was used. One‐way ANOVA analysis with Tukey's post hoc test was performed for comparing ≥3 groups. The Kaplan‐Meier method and log‐rank test were used to determine the survival rate of patients with CRC or AOM/DSS‐induced CRC mice. *p*<0.05 was considered to indicate a statistically significant value.

## Conflict of Interest

The authors declare no conflict of interest.

## Supporting information

Supporting Information

## Data Availability

The data that support the findings of this study are available from the corresponding author upon reasonable request.
