# Supplementary material for: GART Functions as a Novel Methyltransferase in the RUVBL1/β‐Catenin Signaling Pathway to Promote Tumor Stemness in Colorectal Cancer
Source: Adv Sci (Weinh). 2023 Jul 13;10(25):2301264. doi: 10.1002/advs.202301264 (PMC10477903; doi:10.1002/advs.202301264)
Supplement: Supplementary file 1 — Supporting Information [file ADVS-10-2301264-s001.pdf]

ADVANCED SCIENCE
Open Access

Supporting Information

for *Adv. Sci.*, DOI 10.1002/advs.202301264

GART Functions as a Novel Methyltransferase in the RUVBL1/β-Catenin Signaling Pathway to Promote Tumor Stemness in Colorectal Cancer

Chao Tang, Mengying Ke, Xichao Yu, Shanliang Sun, Xian Luo, Xin Liu, Yanyan Zhou, Ze Wang, Xing Cui, Chunyan Gu* and Ye Yang**

Supplementary Materials and Methods

Patients. Patients were considered eligible for this study if they were histologically diagnosed with metastatic colorectal cancer (CRC). CRC patients had received second-line standard chemotherapy, with progression and/or recurrence before enrollment. Pemetrexed (PEM) was evaluated as a third or fourth-line treatment. Other criteria for eligibility included measurable disease by the Response Evaluation Criteria in Solid Tumors, an Eastern Cooperative Oncology Group performance status of ≤2, adequate blood counts (neutrophils ≥1500/mm^3 and platelets ≥1x10^5/mm^3), renal function within normal limits, total bilirubin ≤1.5 mg/dL, and transaminases ≤2.5 times the upper limit of normal.

Treatment. PEM treatment study in CRC patients were conducted by Dr. Cui Xing in the Second Affiliated Hospital of Shandong University of Traditional Chinese Medicine. A total of 12 patients received PEM monotherapy. The dose of PEM was 500 mg/m^2 at Day 1, and the dose of other chemotherapy drugs was calculated based on the body surface area of patients according to the recommendation of NCCN guidelines. Each cycle lasted for 21 d.

Evaluation. Baseline tumor measurements by computed tomography were obtained within 28 d before starting study treatment. Physical examinations, including medical history, laboratory studies, and assessment of performance status, were conducted at the beginning of each 3-week cycle.

Tumor response was evaluated every 2 cycles by computed tomography imaging and tumor measurement done using the Response Evaluation Criteria in Solid Tumors (RECIST) 1.1 criteria.[1]

Tumor response included complete response (CR), partial response (PR), stable disease (SD) and progression disease (PD). All toxicities were graded according to the National Cancer Institute's Common Toxicity Criteria for adverse events, version 5.0 (CTCAE 5.0). Retreatment at the start of each

cycle required adequate hematologic function (absolute neutrophil count ≥1500/mm^3 and platelets ≥1x10^5/mm^3) and resolution of all toxicities to ≤CTC grade 2.

The treatment was continued until CRC development, unacceptable toxicity, withdrawal of patient consent, or dead.

Statistical analysis. The primary end point of this study was progression-free survival (PFS), and the secondary end points were disease control rate (DCR), objective response rate (ORR), overall survival (OS) and safety.

PFS was defined as the time period from the initiation of PEM based chemotherapy regimens and the first evaluation of RECIST1.1 for solid tumors as disease progression or death from any cause, whichever occurred first. OS was defined as the time from initiation of PEM based chemotherapy regimens to death from any cause or the last follow-up. DCR was defined as the percentage of patients whose tumors had shrunk or remained stable for a certain period of time in the evaluable number of cases, including complete response (CR), partial response (PR), and stable response (SD). ORR referred to the percentage of evaluable cases in which tumor shrinkage had reached a certain level and remained constant, including complete response (CR) and partial response (PR).

PFS and OS times were estimated by the Kaplan-Meier method. Univariate subgroup analyses of age, sex, primary site, degree of differentiation, presence of liver metastases, and baseline CEA level were performed by log-rank test.

Reference

1 Therasse P, Arbuck SG, Eisenhauer EA*, et al.* New guidelines to evaluate the response to treatment in solid tumors. European Organization for Research and Treatment of Cancer, National Cancer Institute of the United States, National Cancer Institute of Canada. *J Natl Cancer Inst*

2000;92:205-16.

Supplementary Figures

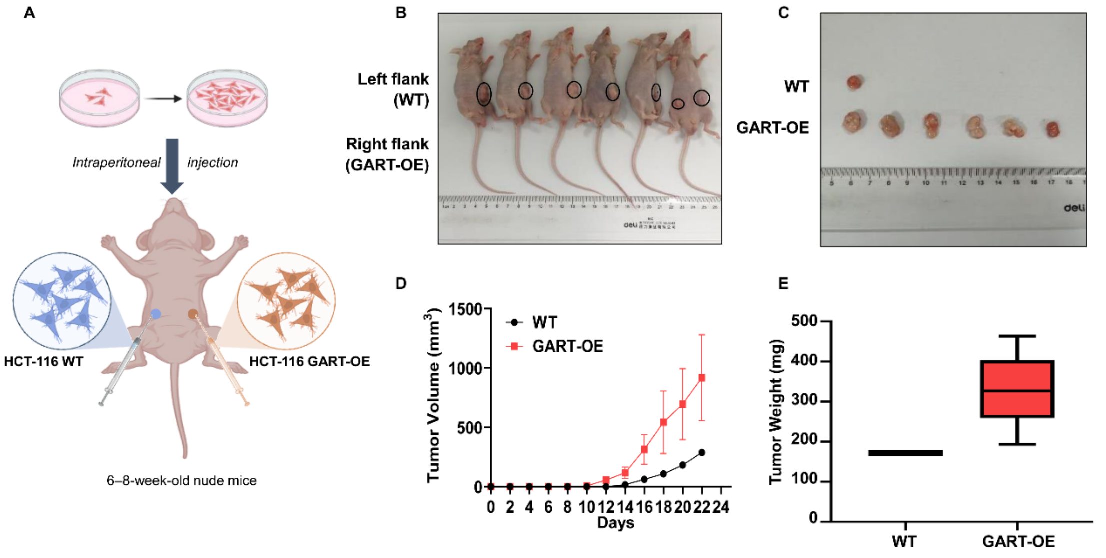

Figure S1. The cell-derived xenograft model. (A) Establishment of CRC subcutaneous xenograft model. (B) Photographic images of xenograft mice at Day 22. (C) Schematic images of xenograft tumors from 6 nude mice. (D) The dynamic change of tumor volume in 6 nude mice. (E) Mean tumor weight in 6 nude mice.

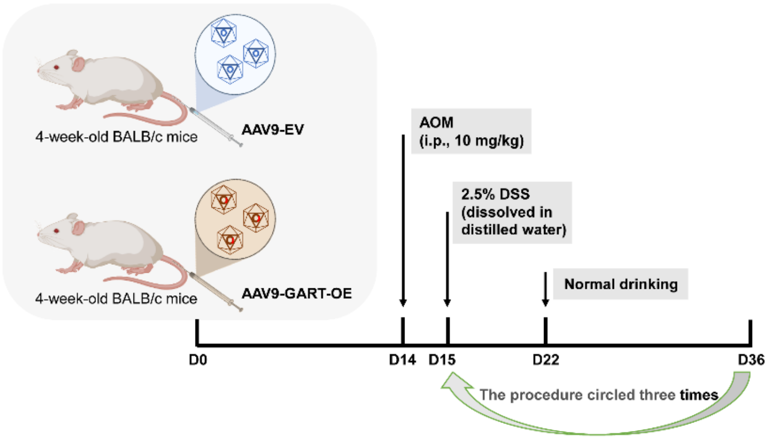

Figure S2. Schematic diagram of establishing the azoxymethane (AOM)/dextran sulfate

sodium (DSS)-induced CRC model.

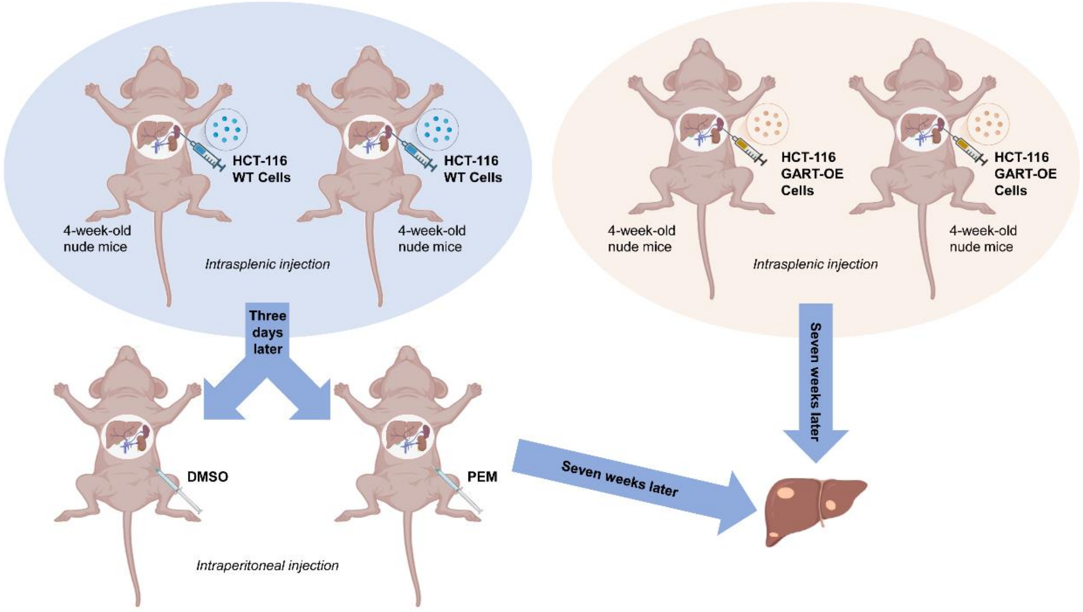

Figure S3. Schematic diagram of establishing the liver metastasis model.

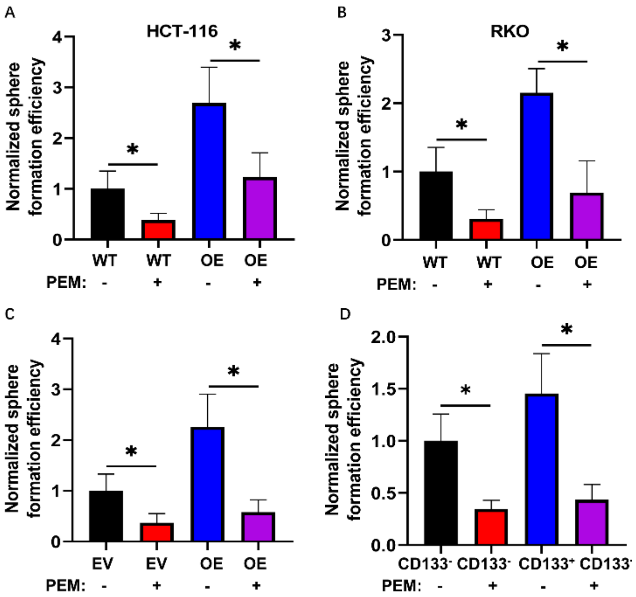

Figure S4. Statistical analysis for Figure 3C-D in the manuscript. (A & B) Statistical analysis for the representative images of formed spheroids derived from WT and

GART-OE cells treated with/without pemetrexed (PEM). (C & D) Statistical analysis for the representative images of spheroids formed from primary mouse and human CRC cells treated with/without PEM. The data are expressed as the mean ± SD. *p<0.05.

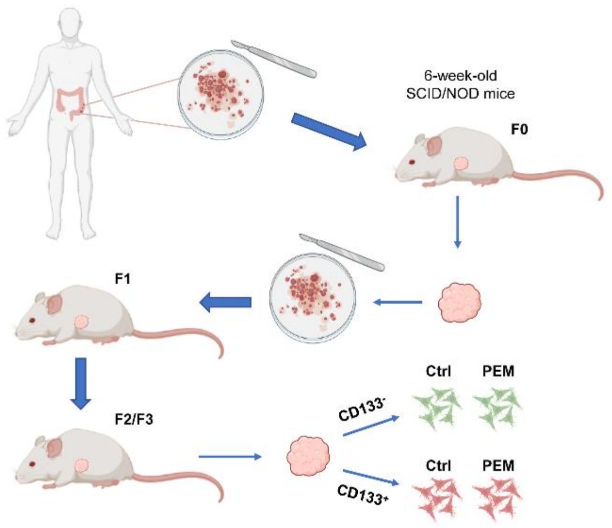

Figure S5. Schematic diagram of establishing the CRC patient-derived tumor xenograft (PDX) model.

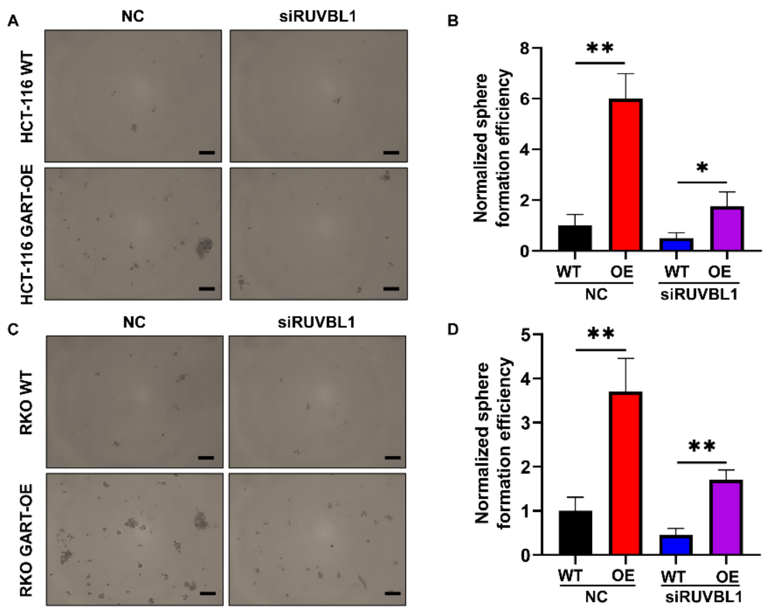

Figure S6. RUVBL1 participates in GART-mediated CRC cell stemness. (A & B)

Representative images and statistical results of spheroids formed by HCT-116 WT and GART-OE cells interfered with NC or RUVBL1 siRNAs. Scale bar = 200 μm. (C & D) Representative images and statistical results of spheroids formed by RKO WT and GART-OE cells interfered with NC or RUVBL1 siRNAs. Scale bar = 200 μm. The data are expressed as the mean ± SD. *p<0.05, **p<0.01.

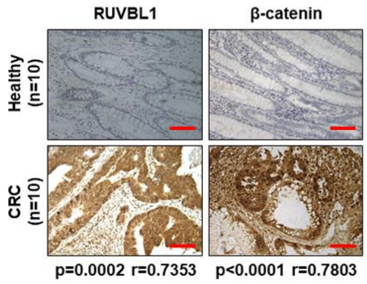

Figure S7. IHC results indicate that RUVBL1 and β-catenin are highly expressed in CRC patient samples. Ki67 was shown in Figure 1T of the manuscript. Scale bar = 50 μm.

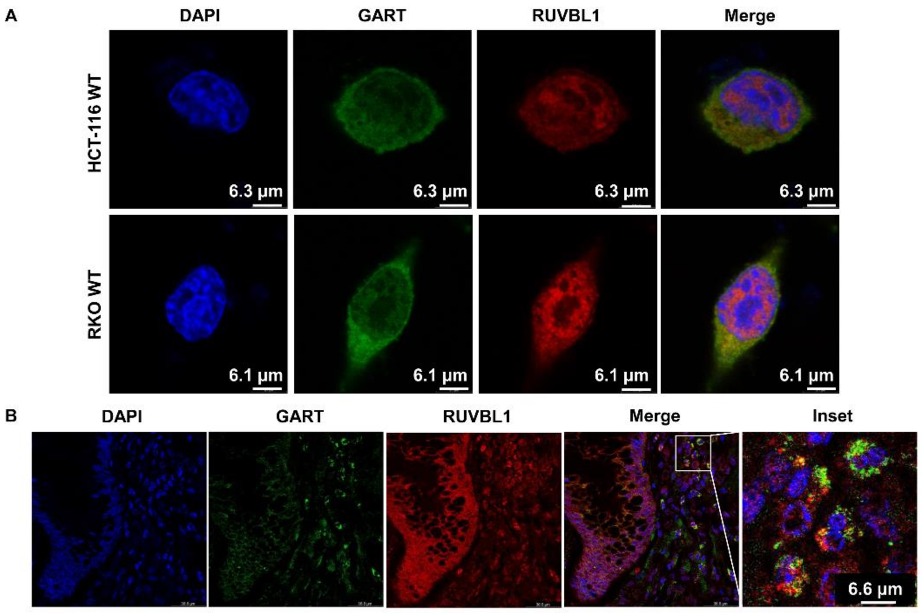

Figure S8. IF assays confirm that GART is co-located with RUVBL1 in both of cytoplasm and nucleus. (A) IF staining of GART and RUVBL1 in HCT-116 and RKO WT cells. (B) IF

staining of GART and RUVBL1 in CRC patient tissues. Nuclei were stained with DAPI (blue).

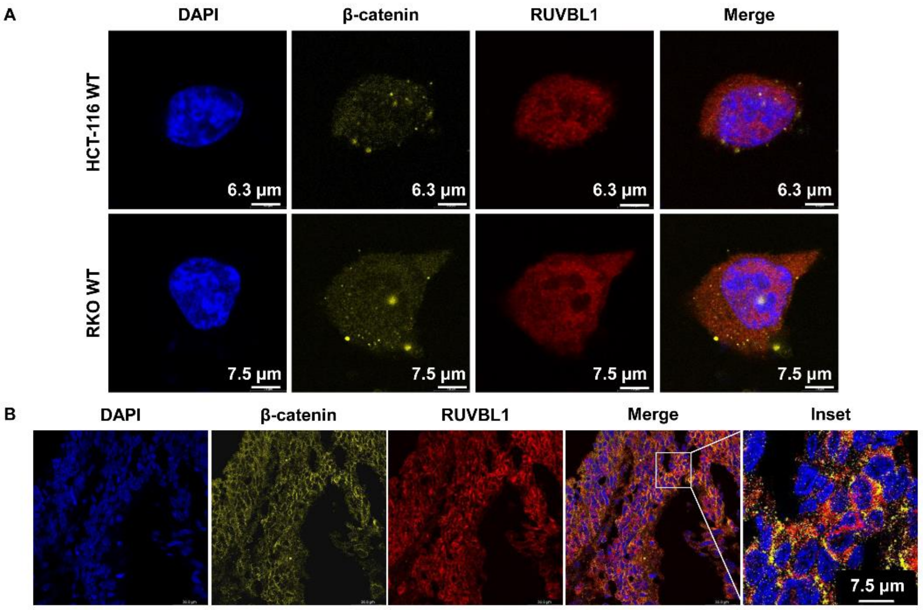

Figure S9. IF assays indicate RUVBL1 is co-located with β-catenin in both of cytoplasm and nucleus. (A) IF staining of RUVBL1 and β-catenin in HCT-116 and RKO WT cells. (B) IF staining of RUVBL1 and β-catenin in CRC patient tissues. Nuclei were stained with DAPI (blue).

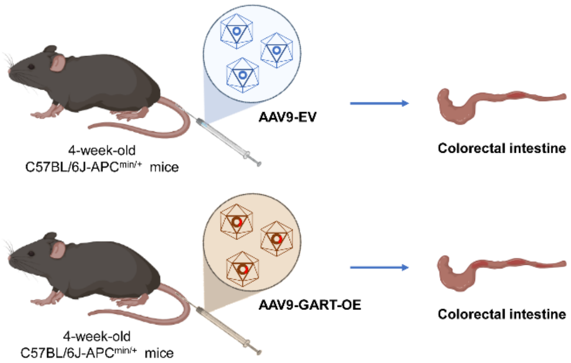

Figure S10. Schematic diagram of the APC$^{min/+}$ mouse-induced CRC model.
